# Sterol Synthesis in Diverse Bacteria

**DOI:** 10.3389/fmicb.2016.00990

**Published:** 2016-06-24

**Authors:** Jeremy H. Wei, Xinchi Yin, Paula V. Welander

**Affiliations:** Department of Earth System Science, Stanford UniversityStanford, CA, USA

**Keywords:** lipid biosynthesis, sterols, biomarkers, squalene epoxidase, oxidosqualene cyclase, myxobacteria, methanotrophs, planctomycetes

## Abstract

Sterols are essential components of eukaryotic cells whose biosynthesis and function has been studied extensively. Sterols are also recognized as the diagenetic precursors of steranes preserved in sedimentary rocks where they can function as geological proxies for eukaryotic organisms and/or aerobic metabolisms and environments. However, production of these lipids is not restricted to the eukaryotic domain as a few bacterial species also synthesize sterols. Phylogenomic studies have identified genes encoding homologs of sterol biosynthesis proteins in the genomes of several additional species, indicating that sterol production may be more widespread in the bacterial domain than previously thought. Although the occurrence of sterol synthesis genes in a genome indicates the potential for sterol production, it provides neither conclusive evidence of sterol synthesis nor information about the composition and abundance of basic and modified sterols that are actually being produced. Here, we coupled bioinformatics with lipid analyses to investigate the scope of bacterial sterol production. We identified oxidosqualene cyclase (Osc), which catalyzes the initial cyclization of oxidosqualene to the basic sterol structure, in 34 bacterial genomes from five phyla (Bacteroidetes, Cyanobacteria, Planctomycetes, Proteobacteria, and Verrucomicrobia) and in 176 metagenomes. Our data indicate that bacterial sterol synthesis likely occurs in diverse organisms and environments and also provides evidence that there are as yet uncultured groups of bacterial sterol producers. Phylogenetic analysis of bacterial and eukaryotic Osc sequences confirmed a complex evolutionary history of sterol synthesis in this domain. Finally, we characterized the lipids produced by Osc-containing bacteria and found that we could generally predict the ability to synthesize sterols. However, predicting the final modified sterol based on our current knowledge of sterol synthesis was difficult. Some bacteria produced demethylated and saturated sterol products even though they lacked homologs of the eukaryotic proteins required for these modifications emphasizing that several aspects of bacterial sterol synthesis are still completely unknown.

## Introduction

Sterols are tetracyclic triterpenoid lipids that are required by all eukaryotes for critical cellular functions including maintaining membrane fluidity, phagocytosis, stress tolerance, and cell signaling (Bloch, 1991; Swan and Watson, 1998; Castoreno et al., 2005; Xu et al., 2005; Riobo, 2012). Studies on the biosynthesis of sterols in eukaryotes have revealed a variety of novel biochemical reactions while molecular and cell biological studies have revealed unique regulatory mechanisms and key insights into sterol transport (Dimster-Denk and Rine, 1996; Yang, 2006; Nes, 2011). Geochemists also have an interest in these molecules as they have the potential to function as “molecular fossils” (Summons et al., 2006; Love et al., 2009). Sterols, like many polycyclic triterpenoids, are quite recalcitrant and their degradation products, the steranes, are readily preserved in ancient sediments. Sterane signatures in the rock record date as far back as 1.6 billion years (Brocks et al., 2005) and, based on their distribution in modern eukaryotes, are utilized as biomarkers for the existence of specific eukaryotic organisms at the time of deposition (Peters et al., 2007a,b). Because eukaryotes are the predominant extant producers of sterols and because they require sterols for growth, the use of steranes as biomarkers for eukaryotes seems robust. However, sterol production has been observed in a few bacterial species raising the question as to whether bacterial sterol production is significant for the interpretation of sterane signatures (Volkman, 2003, 2005).

**Figure 1:**
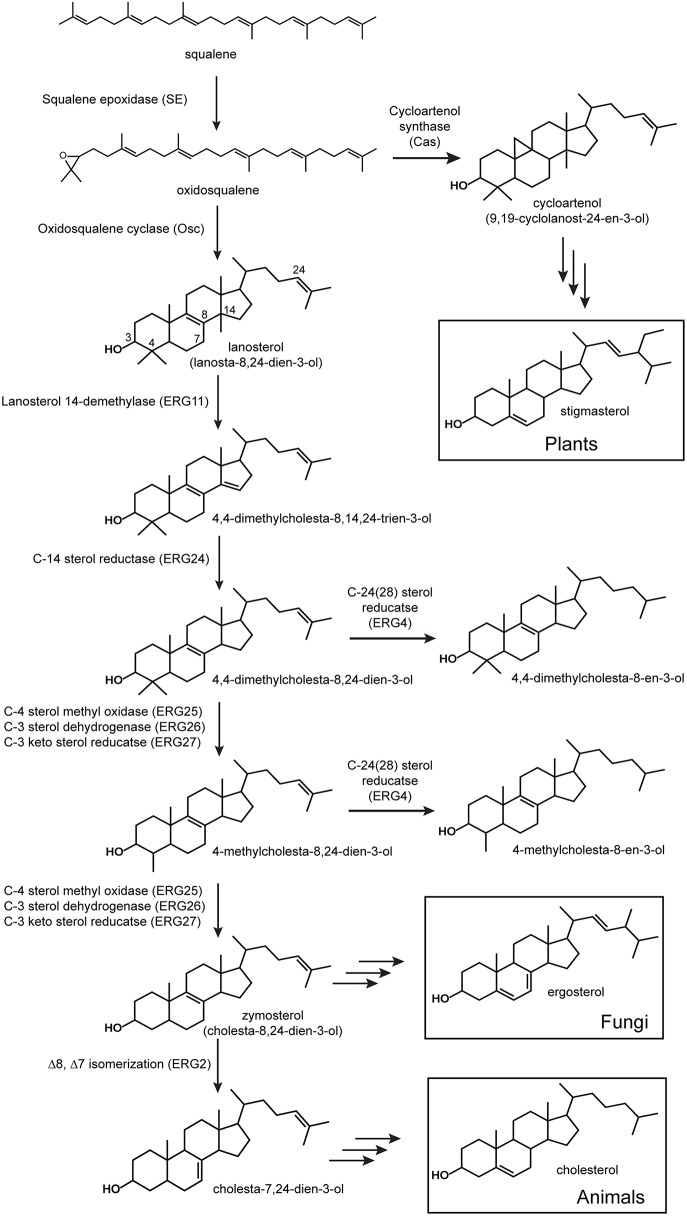
**Sterol biosynthesis in eukaryotes**. All sterol biosynthetic pathways begin with the oxidation of squalene to oxidosqualene and subsequent cyclization to lanosterol (vertebrates and fungi) or cycloartenol (plants). Shown are the initial enzymatic steps in the conversion of lanosterol to zymosterol which occurs similarly in vertebrates and fungi. Proteins involved in these steps have been characterized from a variety of eukaryotes and the locus to tags shown are those from *Saccharomyces cerevisiae* (Erg).

Bacterial sterol production was first discovered in the aerobic methanotroph *Methylococcus capsulatus* Bath (Bird et al., 1971; Bouvier et al., 1976). *M. capsulatus* produces the modified lanosterol products 4,4-dimethylcholesta-8,24-dien-3-ol, 4,4-dimethylcholesta-8-en-3-ol, 4-methylcholesta-8,24-dien-3-ol, 4-methylcholesta-8-en-3-ol (Figure 1). Subsequent studies have demonstrated the production of similar sterols in other aerobic methanotrophs of the Methylococcales order within the γ-Proteobacteria (Schouten et al., 2000; Banta et al., 2015). In addition, sterol biosynthesis has also been observed in a few myxobacteria of the δ-Proteobacteria (Bode et al., 2003) and the planctomycete *Gemmata obscuriglobus* (Pearson et al., 2003). *G. obscuriglobus* produces the least biosynthetically complex sterols, lanosterol and the rare lanosterol isomer parkeol. Two myxobacteria, *Stigmatella aurantica* and *Cystobacter fuscus*, also produce biosynthetically simple sterols but rather than lanosterol they synthesize the cyclopropylsterol cycloartenol which is typically an intermediate in plant sterol synthesis (Figure 1; Bode et al., 2003). However, some myxobacteria do modify their sterol products. In particular, *Nannocystis excedens* produces cholest-7-en-3-ol (lathosterol) and cholest-8-en-3-ol (Bode et al., 2003). Recently, bioinformatics analyses of bacterial genomes revealed sterol biosynthesis genes in bacteria that have yet to undergo lipid analysis (Desmond and Gribaldo, 2009; Villanueva et al., 2014). Desmond and Gribaldo proposed that the myxobacterium *Plesiocystis pacifica* had the genetic potential to produce cholesta-7,24-dienol-3β-ol. In addition, Villanueva et al. observed putative oxidosqualene cyclase (Osc) homologs, required for the initial cyclization of oxidosqualene to lanosterol or cycloartenol (Figure 1) in a variety of bacterial genomes including three aerobic methanotrophs, two Bacteriodetes species, and one cyanobacterium symbiont.

Given the sporadic and sparse distribution of sterol synthesis in the bacterial domain, it has been suggested that bacteria most likely acquired this biosynthetic pathway through horizontal gene transfer from an ancient eukaryotic source (Bode et al., 2003; Pearson et al., 2003; Summons et al., 2006). Recent phylogenetic studies of sterol synthesis proteins have begun to indicate a potentially more complicated ancestry (Desmond and Gribaldo, 2009; Frickey and Kannenberg, 2009; Villanueva et al., 2014). Sterol synthesis in eukaryotes is typically divided into two main biosynthetic pathways defined by the Osc utilized in the initial cyclization reaction (Figure 1; Pearson et al., 2003; Summons et al., 2006; Desmond and Gribaldo, 2009). The lanosterol synthase (LAS) route involves the cyclization of oxidosqualene to lanosterol and leads to the production of cholesterol in vertebrates and ergosterol in fungi (Figure 1; Pearson et al., 2003; Summons et al., 2006; Desmond and Gribaldo, 2009). The cycloartenol synthase (CAS) pathway is considered primarily a plant sterol pathway and is characterized by the conversion of oxidosqualene to cycloartenol by CAS (Pearson et al., 2003; Summons et al., 2006; Desmond and Gribaldo, 2009). As described above, lipid analyses have shown that both of these sterol pathways exist in the bacterial domain. The LAS of *G. obscuriglobus, M. capsulatus*, and *P. pacifica* have been shown to branch basally to eukaryotic Osc sequences suggesting that these bacterial homologs arose through an ancestral lanosterol lineage (Pearson et al., 2003; Desmond and Gribaldo, 2009; Frickey and Kannenberg, 2009). On the other hand, the myxobacterium *S. aurantica*, which produces cycloartenol, has a CAS homolog that is well-separated phylogenetically from other bacterial cyclases and is more closely related to eukaryotic sequences (Frickey and Kannenberg, 2009). This was viewed as strong evidence for the acquisition of CAS by *S. aurantica* through horizontal gene transfer most likely from a plant source (Bode et al., 2003; Pearson et al., 2003; Desmond and Gribaldo, 2009; Frickey and Kannenberg, 2009). However, a more recent phylogenetic reconstruction, which included more bacterial CAS and LAS homologs, shows the *S. aurantica* CAS homolog clustering with other cycloartenol producing myxobacteria and forming a distinct clade separate from other eukaryotic Osc sequences (Villanueva et al., 2014). Thus, the ancestry of bacterial sterol synthesis remains an open question that may become clearer as more bacterial sterol cyclases are discovered.

While genomic and phylogenetic data may provide some clues to the diversity and evolutionary history of the sterol biosynthetic pathway in bacteria, it is important to note that the occurrence of an Osc in a bacterial genome demonstrates the potential to produce sterols but it is not conclusive evidence that sterol production is actually occurring. Also, the presence of Osc is only indicative of the initial cyclization required to produce the most basic sterols and it does not provide any insight into how sterols may be modified in these Osc containing bacteria. Sterol production is not uniform across all eukaryotes both in terms of the final products produced and in the proteins and enzymatic mechanisms involved in their biosynthesis (Hartmann, 1998; Volkman, 2003; Summons et al., 2006). As mentioned above, vertebrates synthesize cholesterol as a final product while fungi generate ergosterol and plants tend to make stigmasterol (Figure 1; Desmond and Gribaldo, 2009). Downstream modifications in eukaryotes, including methylations, unsaturation, and isomerization (Kodner et al., 2008; Desmond and Gribaldo, 2009), also differ and it is unclear whether sterol biosynthesis in bacteria is more similar to one (or none) of these pathways.

To fully understand both the diversity of sterol production in the bacterial domain and the evolutionary history of the sterol biosynthetic pathway, further studies are needed to characterize sterol production in bacterial species. In this study, we identify potential sterol biosynthesis genes in a variety of bacterial genomes and metagenomes. We also characterize the lipid profiles of a subset of these potential sterol-producers and demonstrate that all but one of the organisms we tested were capable of sterol production under laboratory conditions. Through these studies, it is evident that sterol production is more widespread in the bacterial domain than previously thought and that the bacterial sterol biosynthetic pathway has a complex evolutionary history.

## Materials and methods

### Bioinformatics analyses

Homologs of the *M. capsulatus* Osc protein (locus tag: MCA2873) were identified through BLASTP (Altschul et al., 1997) searches of all bacterial (31,237) and eukaryotic (220) genomes as well as all environmental metagenomes (2707) on the Joint Genome Institute Integrated Microbial Genomes database (http://img.jgi.doe.gov/). MUSCLE (Edgar, 2004) alignments of bacterial and eukaryotic Osc sequences with an e-value of 1e-50 or lower were generated in Geneious (Biomatters). For metagenomic sequences, only Osc candidates larger than 400 amino acids were included in the alignment. Redundancy in metagenomic alignments was decreased utilizing the Decrease Redundancy web tool (http://web.expasy.org/decrease_redundancy/). Due to large gaps in the metagenomic alignments it was also necessary to extract conserved regions with GBLOCKS (Talavera and Castresana, 2007) prior to building phylogenetic trees containing metagenomics sequences. PhyML (Guindon and Gascuel, 2003) was utilized to generate maximum likelihood phylogenetic trees using the LG+gamma model, four gamma rate categories, ten random starting trees, NNI branch swapping, and substitution parameters estimated from the data. Resulting phylogenetic trees were edited in the Interactive Tree of Life (iTOL) website (http://itol.embl.de/; Letunic and Bork, 2007, 2011, 2016).

The following yeast and bacterial proteins were used to search bacterial genomes (BLASTP) for homologs of other sterol biosynthesis proteins: squalene epoxidase (*M. capsulatus* locus tag: MCA2872); lanosterol 14-α-demethylase ERG11 (*Saccharomyces cerevisiae* locus tag: YHR007C); C-14 sterol reductase ERG24 (*S. cerevisiae* locus tag: YNL280C); C-4 methyl sterol oxidase ERG25 (*S. cerevisiae* locus tag: YGR060W); C-3 sterol dehydrogenase ERG26 (*S. cerevisiae* locus tag: YGL001C); 3-keto sterol reductase ERG27 (*S. cerevisiae* locus tag: YLR100W); C-24(28) sterol reductase ERG4 (*S. cerevisiae* locus tag: YGL012W0; 24-dehydrocholesterol reductase (*Homo sapiens* locus tag: HGNC:2859). The e-value cut-off for a potential homolog of these proteins was set at 1e-10 or lower with a minimum 20% identity.

### Lipid analyses

Bacterial strains surveyed for sterol production and their growth conditions are described in Table 1. None of the media utilized for growth contained yeast extract. All strains were grown in our laboratory except for *Enhygromyxa salina* DSM15201, *Plesiocystis pacifica* SIR-1 DSM14875, and *Sandaracinus amylolyticus* DSM53668. For lipid analysis of these three strains, cells were scraped directly from the agar plates purchased from the German Collection of Microorganisms and Cell Cultures (DSMZ;), placed in 2 ml of deionized water and stored at −20°C. All liquid cultures were centrifuged at 5000 × g for 10 min at 4°C and the supernatant was discarded. Cell pellets were frozen at −20°C prior to lipid extraction.

**Table 1:** **Bacterial strains tested for sterol biosynthesis**.

**Bacteria surveyed in this study**	**Growth conditions**	**Source**
*Corallococcus coralloides;* DSM 2259	DSMZ medium 222 at 30°C with shaking at 200 rpm; lipid analysis from 50 ml of a stationary phase culture	DSMZ
*Cystobacter fuscus;* DSM 2262	DSMZ medium 222 at 30°C with shaking at 200 rpm; lipid analysis from 50 ml of a stationary phase culture	DSMZ
*Enhygromyxa salina;* DSM 15201	DSMZ medium 958 on agar plates at 30°C; lipid analysis from cells scraped off agar plates	DSMZ
*Fluviicola taffensis* RW262; DSM 16823	DSMZ medium 1 for liquid at 30°C with shaking at 200 rpm; lipid analysis from 50 ml of a stationary phase culture	DSMZ
*Methylobacter luteus;* IMV-B-3098	NMS medium (Welander and Summons, 2012) plus methane at 5 psi over ambient at 30°C with shaking at 200 rpm; lipid analysis from 50 ml of a stationary phase culture	M.G. Kalyuzhnaya, San Diego State University
*Methylobacter whittenburyi;* ACM 3310	NMS medium (Welander and Summons, 2012) plus methane at 5 psi over ambient at 30°C with shaking at 200 rpm; lipid analysis from 50 ml of a stationary phase culture	M.G. Kalyuzhnaya, San Diego State University
*Methyloceanibacter caenitepidi;* DSM 27242	DSMZ medium 1488 plus 1% methanol at 37°C with shaking at 200 rpm; lipid analysis from 50 ml of a stationary phase culture	M.G. Kalyuzhnaya, San Diego State University
*Methylococcus capsulatus* Texas; ATCC 19069	NMS medium (Welander and Summons, 2012) plus methane at 5 psi over ambient at 30°C with shaking at 200 rpm; lipid analysis from 50 ml of a stationary phase culture	ATCC
*Methylosarcina lacus;* LW14	NMS medium (Welander and Summons, 2012) plus methane at 5 psi over ambient at 30°C with shaking at 200 rpm; lipid analysis from 50 ml of a stationary phase culture	M.G. Kalyuzhnaya, San Diego State University
*Plesiocystis pacifica* SIR-1; DSM 14875	DSMZ medium 958 on agar plates at 30°C; lipid analysis from cells scraped off agar plates	DSMZ
*Sandaracinus amylolyticus*; DSM 53668	DSMZ medium 67 on agar plates at 30°C; lipid analysis from cells scraped off agar plates	DSMZ

Frozen cell pellets were resuspended in 2 ml of deionized water and transferred to a solvent washed Teflon centrifuge tube. Five milliliters of methanol and 2.5 ml of dichloromethane were added and the cell mixture was sonicated for 1 h. Ten milliliters of deionized water and 10 ml of dichloromethane were added to samples after sonication, mixed and stored at −20°C overnight. Samples were centrifuged for 10 min at 2800 × g and the organic layer was transferred to a 40 ml baked glass vial. The total lipid extract was evaporated under N_2_ and derivatized to acetate or trimethylsilyl (TMS) esters. For derivatization, an aliquot of the TLE was treated with 50 ml of pyridine and 50 ml of acetic anhydride to create acetate derivatives, or with 25 ml of pyridne and 25 ml of TMS + 1% N,O-bis(trimethylsilyl)trifluoroacetamide (BSTFA) for trimethylsilyl derivative compounds. Samples were dried under N_2_ after derivatization and resuspended in 50–200 μl of dichloromethane prior to high temperature gas chromatography-mass spectrometry (GC-MS) analysis (Sessions et al., 2013).

**Figure 2:**
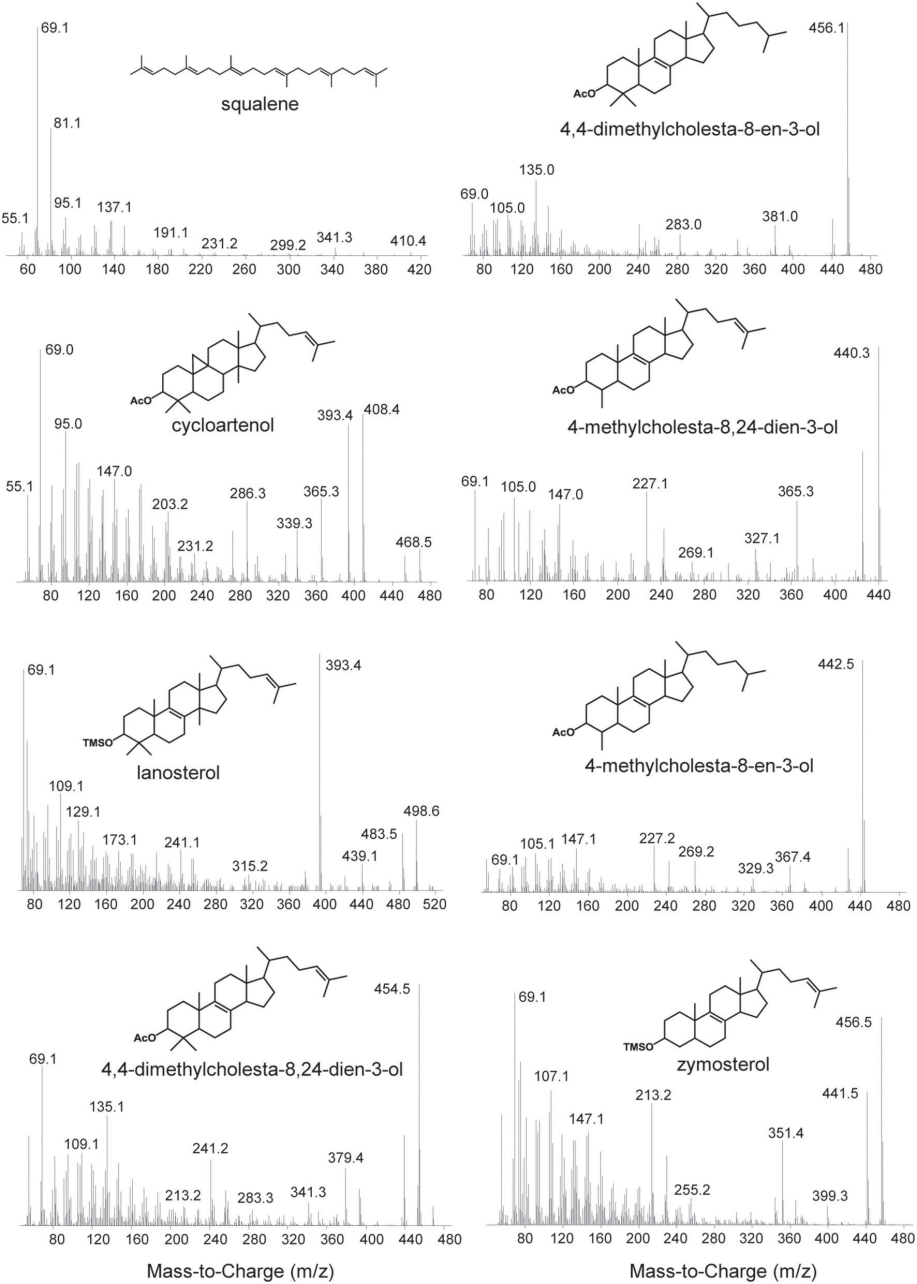
**Mass spectra of sterols identified in this study**. Spectra of the following acetylated sterols: cycloartenol (9,19-cyclolanost-24-en-3-ol), 4,4-dimethylcholesta-8,24-dien-3-ol, 4,4-dimethylcholesta-8-en-3-ol, 4-methylcholesta-8,24-dien-3-ol, 4-methylcholesta-8-en-3-ol. Spectra of the following trimethylsilylated sterols: lanosterol (lanosta-8,24-dien-3-ol) and zymosterol (cholesta-8,24-dien-3-ol).

Lipid extracts were separated on an Agilent 7890B Series GC with helium as the carrier gas at a constant flow of 1.0–1.2 ml/min and programmed as follows: 100°C for 2 min, ramp 15°C/min to 320°C, and hold 28–30 min. Analyses were done on a DB5-HT column (30 m × 0.25 mm i.d. × 0.1 μm film thickness) or a DB17-HT column (30 m × 0.25 mm i.d. × 0.15 μm film thickness). Two microliters of the sample were injected into a Gerstel-programmable temperature vaporization (PTV) injector, operated in splitless mode at 320°C. The GC was coupled to a 5977A Series MSD with the source at 230°C and operated at 70 eV in EI mode scanning from 50 to 850 Da in 0.5 s. All lipids were identified based on their retention time and mass spectra (Figure 2) as well as comparison to prepared internal standards and previously published spectra.

## Results

### Identification of oxidosqualene cyclase homologs in genomes and metagenomes

To identify potential bacterial sterol producers, we queried all bacterial isolate genomes in the Joint Genome Institute Integrated Microbial Genomes database (http://img.jgi.doe.gov/) for homologs of the *M. capsulatus* Bath Osc (locus tag: MCA2873). BLASTP analysis recovered 34 bacterial Osc homologs in five different bacterial phyla with an e-value equal to or lower than e^−100^ and greater than 30% similarity (Table 2). As expected, Osc homologs are found in the genomes of five organisms that have been previously shown to produce sterols (Table 2). The myxobacterium *Corallococcus coralloides* also contains an Osc homolog, however, a previous study of myxobacterial species did not detect any sterols in this bacterium (Bode et al., 2003). Prior phylogenetic studies have also identified Osc homologs in the genomes of *P. pacifica* (Desmond and Gribaldo, 2009)*, Eudoraea adriatica, Fluviicola taffensis, Methylobacter marinus, Methylomicrobium buryatense*, and *Methylomicrobium alcaliphilum* (Villanueva et al., 2014) which we also observed here. However, with the exception of *M. alcaliphilum* (Banta et al., 2015), lipid analysis of these species have not been undertaken to verify sterol production. *Prochloron didenmi* is a cyanobacterial obligate symbiont of the marine ascidian *Lissoclinum patella*. *P. didenmi* has not been isolated in pure culture but partial genome sequencing of this symbiont has previously revealed an Osc homolog and lanosterol has been observed in whole ascidian extracts (Donia et al., 2011). Our bioinformatics analysis also detected Osc homologs in strains not previously shown to produce sterols including three myxobacteria, ten Methylococcales, two Cyanobacteria, one α-Proteobacterium (*Methyloceanibacter caenitepidi*), and one Verrucomicrobia (*Verrucomicrobiaceae bacterium)*.

**Table 2:** **Bacterial genomes that contain oxidosqualene cyclase homologs**.

**Genome**	**Locus tag**	**Isolation/Environment**
**δ-PROTEOBACTERIA (MYXOBACTERIA)**		
*Corallococcus coralloides* DSM 2259	COCOR_01777	Soil, Canada (Huntley et al., 2012)
*Cystobacter fuscus* DSM 2262	D187_003104	Soil, Canada (McCurdy, 1969)
*Enhygromyxa salina* DSM 15201	Ga0055550_114516	Marine, intertidal zone (Iizuka et al., 2003b)
*Labilithrix luteola* DSM 27648	AKJ09_09404	Soil, Yakushima Island (Yamamoto et al., 2014)
*Nannocystis exedens* ATCC 25963	Ga0008035_02275	Soil, Desert (Reichenbach, 1970)
*Plesiocystis pacifica* SIR-1	PPSIR1_04883	Marine, intertidal zone (Iizuka et al., 2003a)
*Sandaracinus amylolyticus* DSM 53668	Ga0055546_18051	Soil, India (Mohr et al., 2012)
*Stigmatella aurantiaca* DW4/3-1	STAUR_5418	Soil (Huntley et al., 2011)
**γ-PROTEOBACTERIA (METHYLOCOCCALES)**		
*Methylobacter luteus* IMV-B-3098	MetluDRAFT_1255	Sewage (Bowman et al., 1993)
*Methylobacter* sp. BBA5.1	EK22DRAFT_03359	Environmental, JGI
*Methylobacter whittenburyi* ACM 3310	GY38DRAFT_3867	Terrestrial (Hamilton et al., 2015)
*Methylobacterium marinus* A45	MetmaDRAFT_3943	Marine water column, Framvaren Fjord, Norway (Strand and Lidstrom, 1984)
*Methylocaldum* sp. 175	JC06DRAFT_3873	Environmental, JGI
*Methylocaldum szegediense* O-12	MetszDRAFT_3769	Hot spring (Bowman, 2014)
*Methylococaceae* sp. 73a	EK23DRAFT_02566	Environmental, JGI
*Methylococcus capsulatus* Bath	MCA2873	Hot Spring (Whittenbury et al., 1970)
*Methylococcus capsulatus* Texas (ATCC 19069)	H156DRAFT_2530	Sewer sludge (Kleiveland et al., 2012)
*Methylomicrobium alcaliphilum* 20Z	MEALZ_0768	Sediment, Tuva soda lakes (Khmelenina et al., 1997)
*Methylomicrobium buryatense* 5G	METBUDRAFT_4052	Sediment, Transbaikal soda lakes (Kaluzhnaya et al., 2001)
*Methylomicrobium kenyense* AMO1	IQ34DRAFT_3157	Surface sediment Kenyan soda lake (Kalyuzhnaya et al., 2008)
*Methylosarcina lacus* LW14	MetlaDRAFT_0845	Sediment, Lake Washington (Kalyuzhnaya et al., 2005)
**α-PROTEOBACTERIA (RHIZOBIALES)**		
*Methyloceanibacter caenitepidi* str. Gela4	Ga0077927_11952	Marine sediment hydrothermal vent (Takeuchi et al., 2014)
**BACTERIODETES**		
*Eudoraea adriatica* DSM 19308	G504DRAFT_2316	Coastal waters, Adriatic Sea (Alain et al., 2008)
*Eudoraea* sp. SCGC 5358	K485DRAFT_00205	Marine, North Sea, JGI
*Eudoraea* sp. SCGC 5441	K506DRAFT_00376	Marine, North Sea, JGI
*Eudoraea* sp. SCGC 5444	K507DRAFT_00676	Marine, North Sea, JGI
*Fluviicola taffensis* RW262 DSM 16823	Fluta_3214	Freshwater, River Taff, (O'sullivan et al., 2005)
**CYANOBACTERIA**		
*Planktothrix* sp. st147	st147_cleanDRAFT_00043800	Freshwater, Lake Langersee
*Prochloron didemni* P2-Fiji	Ga0040003_100917	Tunicate symbiont (*Lissoclinum patella*; Donia et al., 2011)
*Prochloron didemni* P3-Solomon	Ga0040004_00276	Tunicate symbiont (*Lissoclinum patella*; Donia et al., 2011)
*Prochloron didemni* P4-Papua New Guinea	Ga0040005_03818	Tunicate symbiont (*Lissoclinum patella*; Donia et al., 2011)
*Westiella intricata* UH HT-29-1	HT291_04925	Soil, Moen Island (Stratmann et al., 1994)
**PLANCTOMYCETE**		
*Gemmata obscuriglobus* UQM 2246	GobsU_010100006605	Freshwater dam, Queensland, Australia
*Gemmata* sp. IIL30	Ga0036985_07173	Environmental, JGI

The 34 bacterial species with Osc homologs in their genomes were isolated from a variety of environments indicating that bacterial sterol producers are not restricted to a specific ecological niche (Table 2). The majority of the myxobacterial sterol producers were acquired from soil environments while two other myxobacterial strains originated from marine ecosystems (McCurdy, 1969; Reichenbach, 1970; Iizuka et al., 2003a,b; Huntley et al., 2011, 2012; Mohr et al., 2012; Yamamoto et al., 2014). The Methylococcales species were enriched from a diverse set of ecological settings including sewage sludge, marine water columns, hot springs, freshwater lake sediments, and soda lake sediments (Whittenbury et al., 1970; Strand and Lidstrom, 1984; Bowman et al., 1993; Khmelenina et al., 1997; Kaluzhnaya et al., 2001; Kalyuzhnaya et al., 2005, 2008; Kleiveland et al., 2012; Bowman, 2014; Hamilton et al., 2015). Several of the other organisms with Osc homologs in their genomes are also from marine and freshwater environments with *M. caenitepidi* originating from a marine hydrothermal vent, *E. adriatica* from coastal sediments of the Adriatic Sea and *F. taffensis* from sediments of the River Taff (O'sullivan et al., 2005; Alain et al., 2008; Takeuchi et al., 2014).

**Figure 3:**
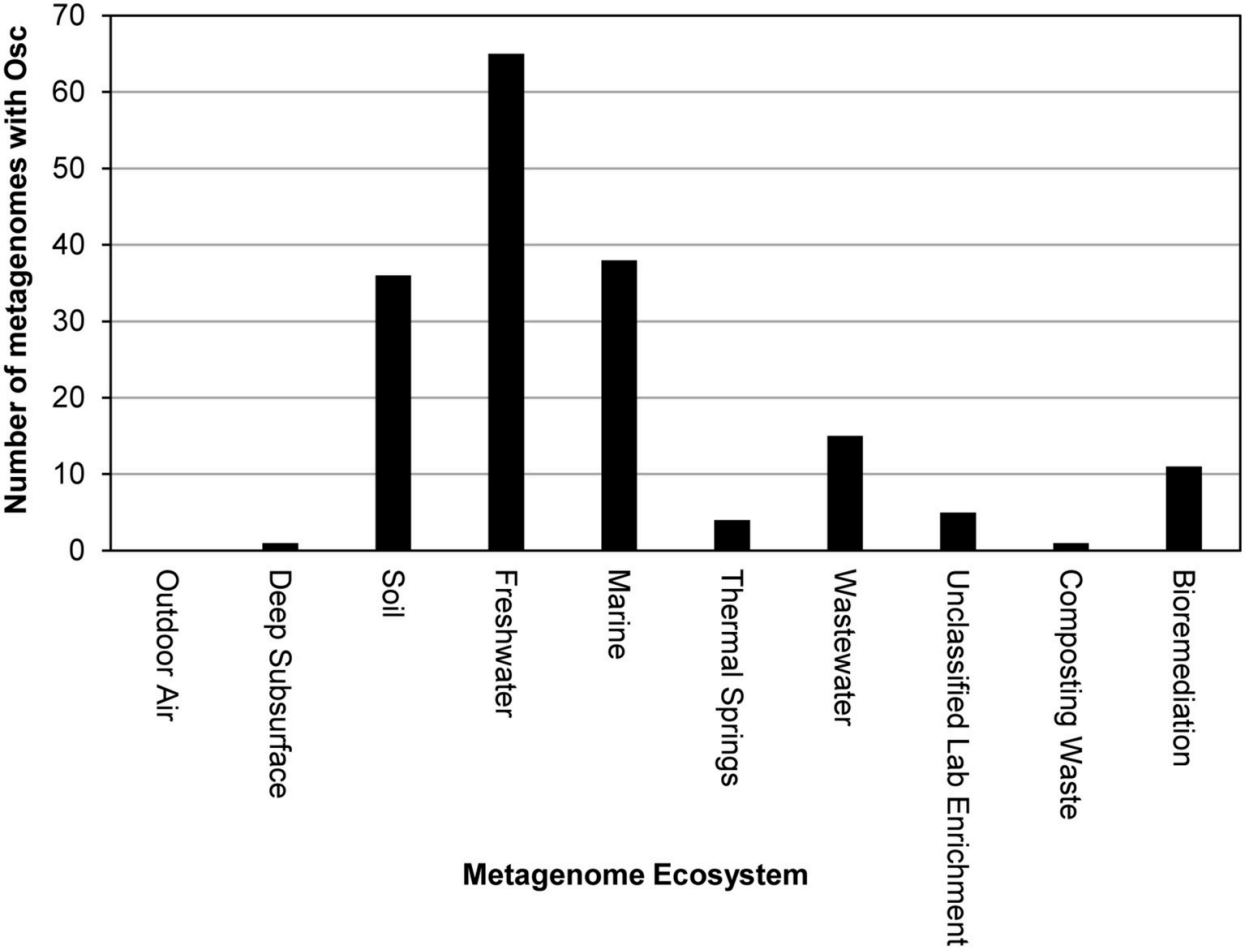
**Distribution of Osc protein sequences in metagenomes**. Each bar represents the number of Osc homologs identified in the metagenomes from that ecosystem. The majority of homologs were found in freshwater, soil and marine metagenomes.

The isolates genomic database utilized in our search represents genomes from organisms that have been cultured and thus can be limited in terms of microbial diversity. We attempted to identify novel Osc homologs potentially from uncultured organisms by performing a BLASTP search of the environmental metagenomics database on the JGI/ IMG website again utilizing the *M. capsulatus* Bath Osc protein as the query sequence. A total of 176 Osc metagenome sequences were identified with an e-value of e^−50^ or lower. The majority of the metagenomic sequences were from soil, marine or freshwater environments similar to the distribution of isolate environments described above (Figure 3). In addition, Osc sequences were found in metagenomes from estuarine microbial mats, hydrothermal vent fluids, and two sequences from sponge symbionts (Figure 3).

### Phylogenetic analysis of genomic and metagenomic Osc sequences

**Figure 4:**
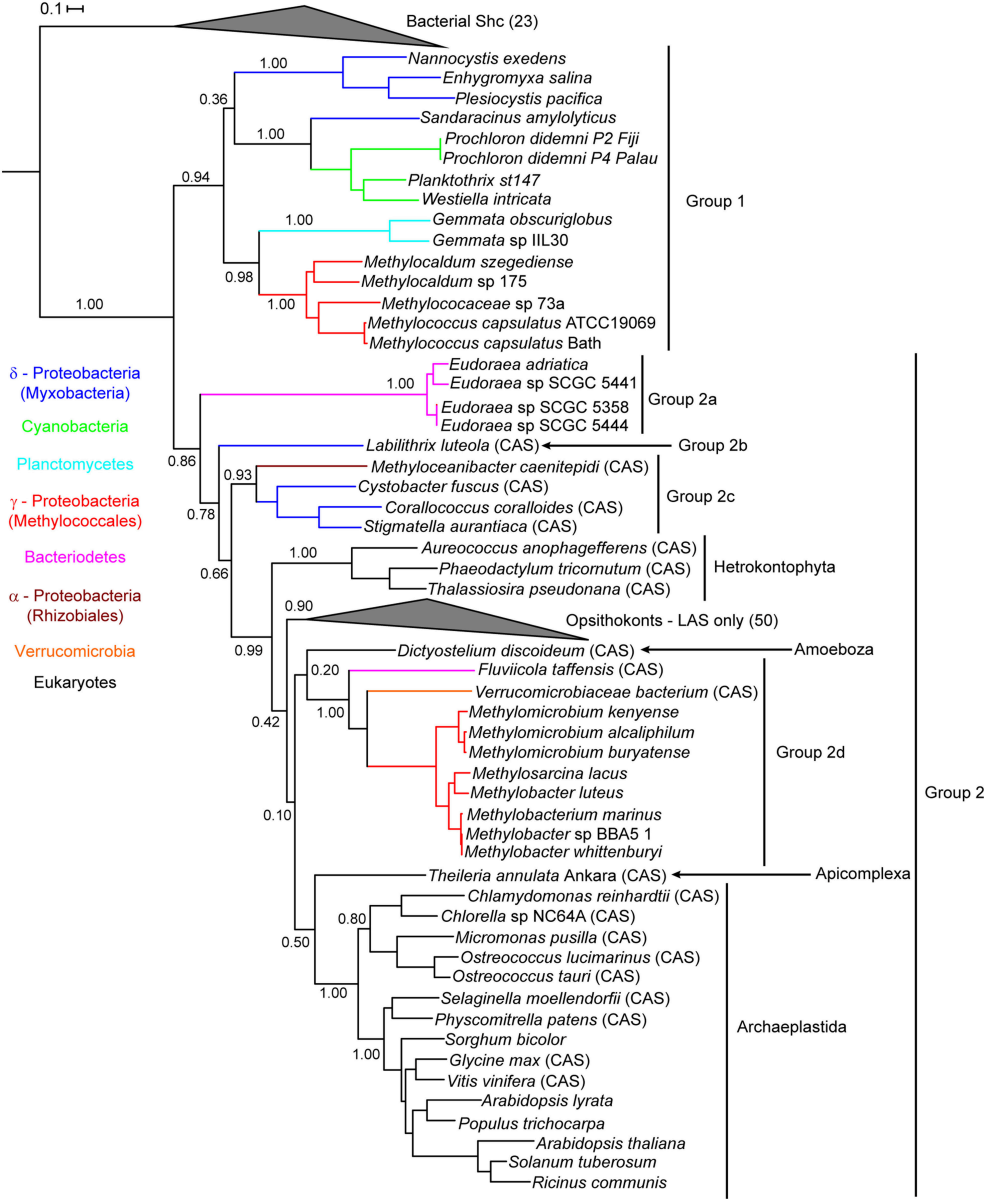
**Maximum likelihood phylogenetic tree of oxidosqualene cyclase protein sequences from bacterial and eukaryotic isolate genomes**. Bacterial squalene hopene cyclase (Shc) sequences were used as the outgroup. Eukaryotic lanosterol synthases (LAS) and bacterial Shc branches are collapsed for better visualization of the tree. Cycloartenol synthases are marked with a CAS following the strain name. Strain names without a CAS label are lanosterol synthase homologs. Colored branches represent different bacterial phyla: δ-Proteobacteria (blue), Cyanobacteria (green), Planctomycetes (cyan), γ-Proteobacteria (red), Bacteriodetes (pink), α-Proteobacteria (brown), and Verrucomicrobia (orange). Black branches represent eukaryotic sequences.

To analyze the phylogeny of the new bacterial Osc homologs identified in our database searches, we generated two maximum likelihood phylogenetic trees. The first tree included only bacterial and eukaryotic Osc sequences obtained from the isolate genomes database which is composed of whole genome sequences from cultured organisms. This phylogenetic tree was created by aligning the 34 bacterial Osc homologs (Table 2) with 70 eukaryotic Osc sequences and 23 bacterial squalene-hopene cyclase (Shc) sequences as the outgroup. Shc catalyze the conversion of squalene to the polycyclic hopanoid diploptene and are structurally and functionally similar to Osc (Siedenburg and Jendrossek, 2011). This tree revealed two lineages of Osc (Figure 4) similar to what was previously observed (Pearson et al., 2003; Desmond and Gribaldo, 2009; Frickey and Kannenberg, 2009; Villanueva et al., 2014). The first clade, Group 1, contains only bacterial LAS sequences (15 total) and branches basally to the eukaryotic cyclases. The second clade, Group 2, includes all eukaryotic CAS and LAS homologs as well as seven bacterial CAS and 12 bacterial LAS homologs (Figure 4). The bacterial sequences within Group 2 can be further classified into various subgroups which seem to be more closely related to each other than to the eukaryotic Osc sequences (Figure 4). Group 2a sequences consists of LAS from *Eudoraea* (Bacteriodetes) species while Group 2b is made up of the one CAS from the myxobacterium *Labilithrix luteola.* Group 2c includes CAS from three myxobacteria and one α-Proteobacterium. The final bacterial subgroup, Group 2d, clusters with the amoeba *Dictyostelium discoideum* CAS and forms a sister clade with the Archaeplastida (plant) cyclases. Group 2d seems to be the one clade to support horizontal gene transfer of a eukaryotic cyclase to bacteria. However, this cluster contains two bacterial CAS and eight bacterial LAS (all Methylococcales) which makes it difficult to ascertain if this lineage originated from a eukaryotic CAS or LAS. Finally, this tree demonstrates that the branching of bacterial Osc sequences is not always congruent with 16S rDNA phylogeny. The Group 1 and Group 2 sequences seem to comprise different lineages yet we observe myxobacterial Osc homologs and aerobic methanotroph sequences in both groups (Figure 4). Also, within Group 2c there is an α-Proteobacterium cyclase clustering with the myxobacterial CAS and the two Bacteriodetes (*Eudoraea* sp. and *F. taffensis*) sequences do not cluster together. Taken together, these observations imply a more complex evolutionary history than previously proposed.

**Figure 5:**
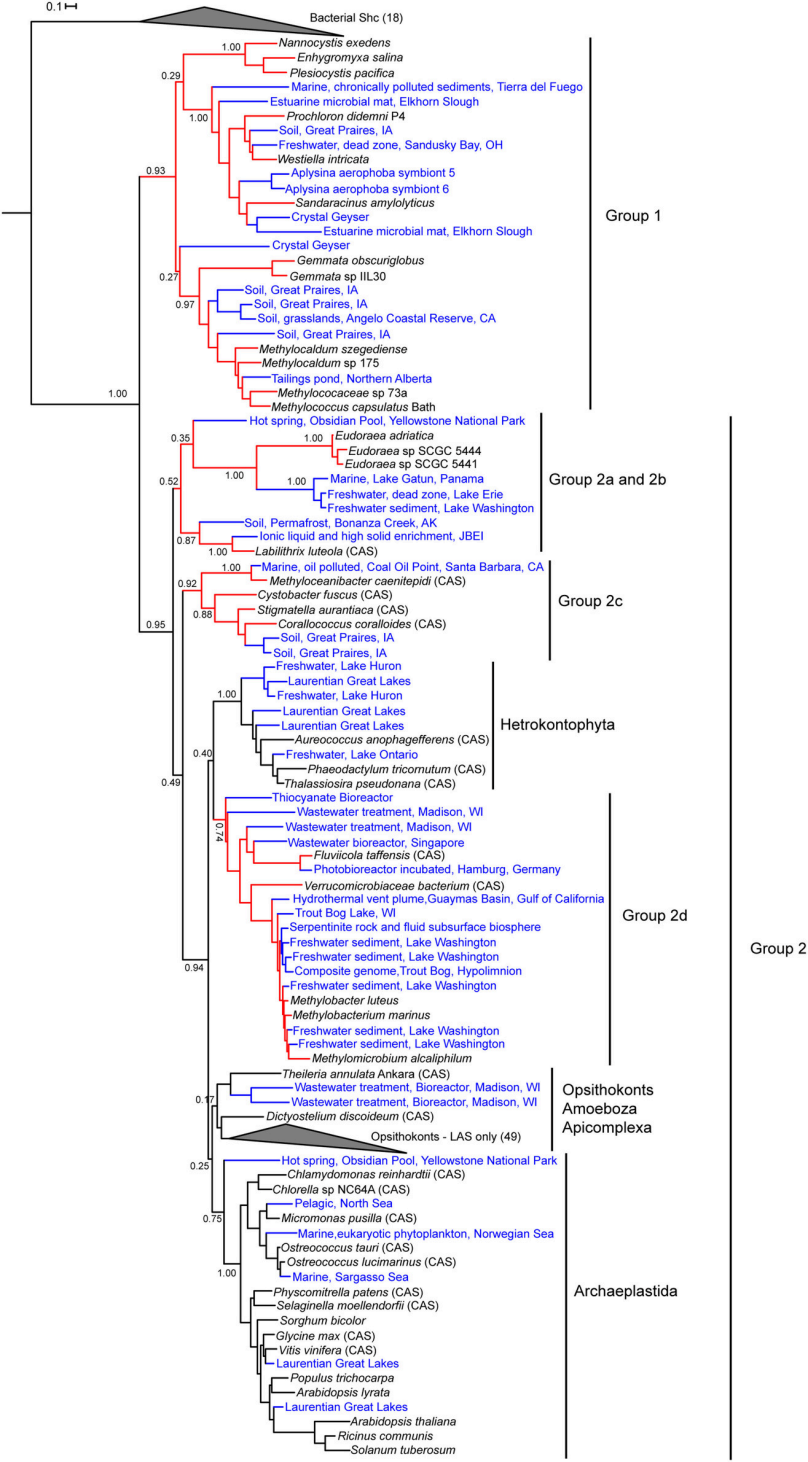
**Maximum likelihood phylogenetic tree of bacterial and eukaryotic genomic and metagenomic Osc protein sequences**. Red branches represent bacterial sequences and black branches are eukaryotic sequences. Blue labels indicate metagenomic sequences and black labels indicate sequences from genomes. Bacterial squalene hopene cyclases (Shc) sequences were used as the outgroup. Eukaryotic lanosterol synthases (LAS) and bacterial Shc branches are collapsed for better visualization of the tree. Cycloartenol synthases are marked with a CAS following the strain name. Strain names without a CAS label are lanosterol synthase homologs.

We constructed a second phylogenetic tree that included the metagenomic Osc sequences identified in our BLAST searches as well as the Osc sequences from cultured organisms used to generate the tree in Figure 4. This metagenomic tree was generated for two reasons: (1) to ascertain if the metagenome Osc sequences identified were from a bacterial or eukaryotic source and (2) to determine if the addition of novel environmental Osc sequences would alter the phylogeny observed in the isolates genome tree (Figure 4). Because many of the sequences retrieved from metagenomes were truncated, we selected 67 of the 176 Osc metagenomic sequences that were at least 400 amino acids (Osc proteins are generally about 600–650 amino acids) to generate a reliable alignment. After reducing redundancy in the alignment, the phylogenetic tree included 55 metagenomic sequences, 65 eukaryotic genomic sequences, and 25 bacterial genomic sequences as well as 18 bacterial Shc sequences as the outgroup. Thirty-seven of the Osc metagenomics sequences retrieved clustered within the bacterial Osc clades (Figure 5) indicating that these sequences were most likely from a bacterial source. Some of these sequences grouped with known sterol producers like the Methylococcales and the myxobacteria. However, some of these metagenomic sequences formed their own clades within the bacterial groups indicating that there are novel sterol-producing bacteria yet to be discovered. Our bioinformatics analysis of metagenomic databases did identify 18 eukaryotic Osc sequences which were related to algal, plant or fungal Osc homologs (Figure 5). Given how widespread sterol synthesis is in eukaryotes, we had expected to detect more eukaryotic Osc sequences than bacterial sequences in metagenomic databases. However, it has been documented that metagenomic sequencing tends to recover few eukaryotic sequences in general (Lindahl and Kuske, 2013). Therefore, the low number of eukaryotic metagenomic sequences more likely reflects a limited number of eukaryotic sequences in metagenome databases rather than the true prevalence of eukaryotic sterol producers in the environment.

The inclusion of metagenomic Osc sequences in the phylogenetic tree did increase the number of homologs in Group 1 but did not significantly alter the branching pattern. However, the addition of more sequences does effect the branching of both bacterial and eukaryotic Osc homologs in Group 2 (Figure 5). In particular, we see that as more bacterial sequences are included, the bacterial Osc homologs form clusters that seem to be more related to each other and distinct from the eukaryotic clades. In this iteration, the *L. luteola* CAS (Group 2b) now clusters in a clade with the *Eudoraea* (Bacteriodetes) LAS (Group 2a) that branches separately from the other Osc sequences in Group 2. Further, the Group 2d sequences no longer cluster with the amoeba *D. discoideum* or form a sister clade with the Archaeplastida. Rather Group 2d forms a sister clade with the hetrokontophyta similar to what was previously observed (Villanueva et al., 2014). Together the Group 2d bacterial CAS and LAS homologs and the hetrokontophyta CAS sequences form a branch that is distinct from all other eukaryotic Osc sequences. Thus, it is possible that this specific bacterial lineage is derived from an ancestral hetrokontophyta CAS as proposed by Villanueva et al. (2014).

### Lipid analysis of potential sterol producers

Our identification of Osc homologs in bacterial genomes demonstrates that the potential for sterol synthesis exists in a variety of bacteria. However, the majority of these potential sterol-producing bacterial strains have not been tested for sterol production. In addition, the occurrence of Osc in a genome only suggests the production of the most basic sterols: lanosterol or cycloartenol. Thus, lipid analysis is needed not just to verify sterol production but also to determine if and how sterols are modified in bacteria. We performed lipid analysis on 11 Osc-containing bacteria that included five myxobacteria, four Methylococcales, one Bacteriodetes, and one α-Proteobacterium (Table 1). In addition, we searched the genomes of these 11 organisms for other sterol biosynthesis protein homologs. Our goal was to link the occurrence of these downstream biosynthesis genes with any sterol modifications, such as saturations and demethylations, these bacteria may be carrying out.

### Sterol production in the myxobacteria

**Figure 6:**
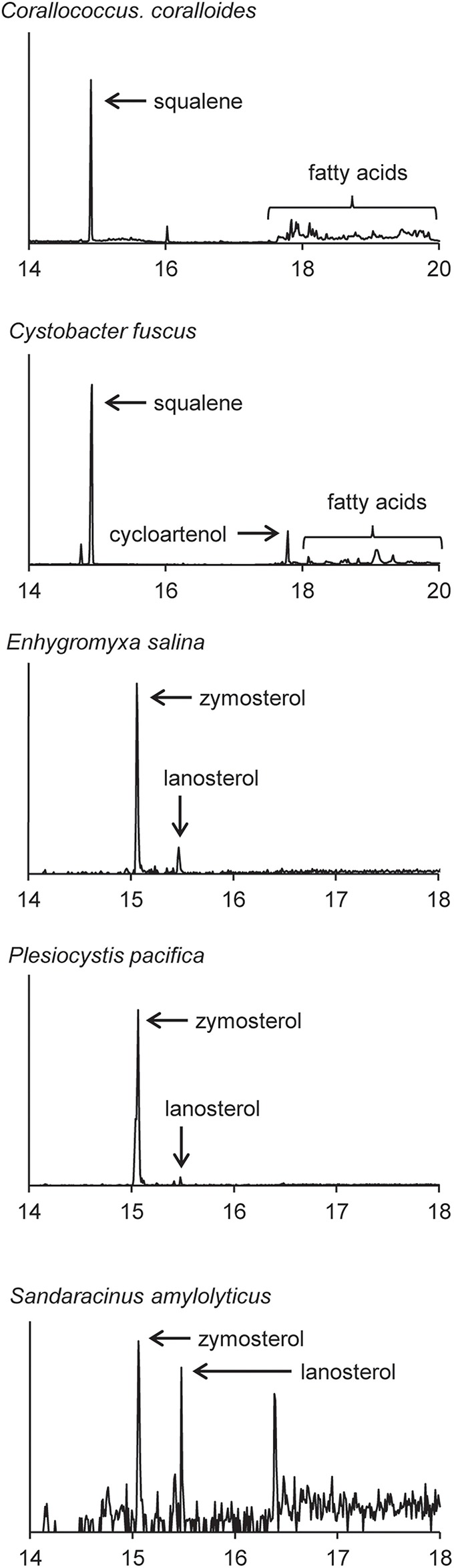
**Sterols production in the myxobacteria**. Extracted ion chromatograms (m/z 69, 440, 442, 454, 456, 468, and 498) of total lipid extract (TLE) from five myxobacteria. *C. coralloides* and *C. fuscus* TLEs were extracted from liquid cultures and were acetylated prior to running on the GC-MS. *E. salina, P. pacifica*, and *S. amylolyticus* TLEs were extracted from cultures on a plate as growing them in liquid cultures was difficult. These TLEs were trimethylsilylated prior to running on the GC-MS. Sterol peaks were identified based on their mass spectra as shown in Figure 2.

Four of the five myxobacterial strains tested were found to produce sterols (Table 3). *C. fuscus* strains were previously reported to produce either lanosterol or cycloartenol (Bode et al., 2003) and the *C. fuscus* strain we analyzed produced cycloartenol. We identified homologs for C-14 demethylation and C-24 reduction in the *C. fuscus* genome but did not observe any sterols with these modifications (Table 4). The other three myxobacteria, *E. salina, P. pacifica*, and *S. amylolyticus* all produced lanosterol rather than cycloartenol and all three strains modified lanosterol to generate zymosterol (cholesta-8,24-dien-3-ol; Figures 1, 6). The conversion of lanosterol to zymosterol requires demethylation at C-4 and C-14 and a reduction at C-14. A previous study had identified homologs for these biosynthetic steps in the *P. pacifica* genome (Desmond and Gribaldo, 2009) and we also observe these protein homologs in *E. salina* and *S. amylolyticus*. However, C-4 demethylation requires three proteins in yeast and vertebrates (ERG25, ERG26, and ERG27) and we do not observe homologs to all of these proteins in these three strains. Desmond et al. pointed out that *P. pacifica* did not have a homolog for one of the three proteins required for C-4 demethylation (ERG27) and we demonstrate that *S. amylolyticus* is also missing this protein. Further, we were only able to identify one homolog of these three proteins in *E. salina* (Table 4). Thus, it is unclear how these myxobacterial strains are fully demethylating at the C-4 position.

**Table 3:** **Sterols identified in bacterial strains**.

	**Squalene**	**Cycloartenol**	**Lanosterol**	**Parkeol**	**4,4-Dimethylcholesta-8, 24-dien-3-ol**	**4,4-Dimethylcholesta-8-en-3-ol**	**4-Methylcholesta-8,24-dien-3-ol**	**4-Methylcholesta-8-en-3-ol**	**Zymosterol**
**MYXOBACTERIA**									
*Corallococcus coralloides*	+								
*Cystobacter fuscus*	+	+							
*Enhygromyxa salina*		+	+						+
*Plesiocystis pacifica*		+	+						+
*Sandaracinus amylolyticus*			+						+
**METHYLOCOCCALES**									
*Methylobacter luteus*						+		+	
*Methylobacter whittenburyi*					+	+	+	+	
*Methylococcus capsulatus* Texas					+	+	+	+	
*Methylosarcina lacus*					+		+		
**BACTERIODETES**									
*Fluviicola taffensis*		+							
**α-PROTEOBACTERIA**									
*Methyloceanibacter caenitepidi*		+							
**STRAINS TESTED IN PREVIOUS STUDIES**									
*Nannocystis* species (Bode et al., 2003)	+		+		+	+	+	+	+
*Stigmatella aurantica* (Bode et al., 2003)	+	+							
*Cystobacter* species (Bode et al., 2003)	+	+	+						
*Corallococcus* species (Bode et al., 2003)	+								
*Gemmata obscuriglobus* (Pearson et al., 2003)			+	+					
*Methylococcus capsulatus* Bath (Bird et al., 1971; Bouvier et al., 1976)					+	+	+	+	
*Methylomicrobium alcaliphilum* 20Z (Banta et al., 2015)						+			

**Table 4:** **Identification of sterol biosynthesis genes in bacterial genomes**.

						**C-4 demethylation**			
	**Squalene oxygenation SE**	**Oxidosqualene cyclization—Las (lanosterol)**	**Oxidosqualene cyclization—Cas (cycloartenol)**	**C-14 demethylation ERG11**	**C-14 reduction ERG24**	**C-4 methyl oxidation ERG25**	**C-3 dehydrogenation ERG26**	**C-3 ketoreduction ERG27**	**C-24(28) reduction ERG4/DHCR24**
**STRAINS TESTED IN THIS STUDY**									
*Corallococcus coralloides*	COCOR_01775		COCOR_01777	COCOR_02429					COCOR_02182
*Cystobacter fuscus*	D187_003106		D187_003104	D187_005870		D187_005391			D187_001522
*Enhygromyxa salina*	Ga0055550_102654	Ga0055550_114516		Ga0055550_103211	Ga0055550_105926	Ga0055550_101078			Ga0055550_101115
*Eudoraea adriatica*	G504DRAF_2315	G504DRAFT_2316							
*Fluviicola taffensis*	Fluta_3221		Fluta_3214						
*Methylobacter luteus*	MetluDRAFT_1256	MetluDRAFT_1255		MetluDRAFT_1253					MetluDRAFT_1263
*Methylobacter whittenburyi*	GY38DRAFT_3868	GY38DRAFT_3867		GY38DRAFT_3865					GY38DRAFT_3875
*Methyloceanibacter caenitepidi*	GL4_3111		GL4_3110						
*Methylococcus capsulatus* Texas	H156DRAF_2531	H156DRAFT_2530		H156DRAFT_1746					H156DRAF_0889
*Methylosarcina lacus*	MetlaDRAFT_0846	MetlaDRAFT_0845		MetlaDRAFT_0843					
*Plesiocystis pacifica*	PPSIR1_02838	PPSIR1_02843		PPSIR1_36894	PPSIR1_23244	PPSIR1_14275	PPSIR1_17435		
*Sandaracinus amylolyticus*	Ga0055546_12562	Ga0055546_18051		Ga0055546_101121	Ga0055546_17127	Ga0055546_103226	Ga0055546_17162		Ga0055546_16346
**STRAINS TESTED IN PREVIOUS STUDIES**									
*Nannocystis excedens*	Ga0008035_04851	Ga0008035_04852		Ga0008035_02418	Ga0008035_01127	Ga0008035_06338	Ga0008035_00875		Ga0008035_06674
*Stigmatella aurantica*	STAUR_5420		STAUR_5418	STAUR_2030		STAUR_2074			
*Gemmata obscuriglobus*	GobsU_010100006610	GobsU_010100006605							
*Methylococcus capsulatus* Bath	MCA2872	MCA2873		MCA2711					MCA1404
*Methylomicrobium alcaliphilum* 20Z	MEALZ_0767	MEALZ_0768		MEALZ_0770	MEALZ_1312				MEALZ_3890

**Figure 7:**
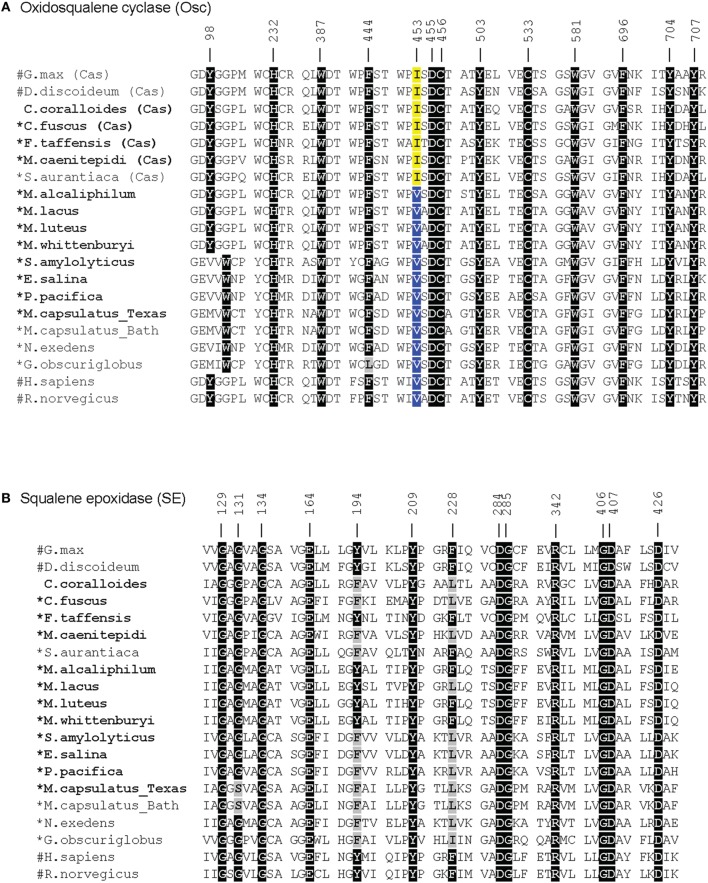
**Amino acid alignments of the critical functional domains of oxidosqualene cyclase (A) and squalene epoxidase (B) homologs adapted from Fischer and Pearson (2007)**. Residues in black indicate residues that have been demonstrated to have a role in the biosynthesis of sterols in eukaryotes (Ruckenstuhl et al., 2005; Abe et al., 2007; Fischer and Pearson, 2007). Gray residues are those that differ from the conserved residue. In the Osc alignment, an isoleucine (I) at 453 (yellow) indicates a cycloartenol synthase and a valine (V) at 453 (blue) indicates a lanosterol synthase (Summons et al., 2006). Numbers correspond to residues in human Osc and SE. Bold labels indicate bacterial strains tested in this study. ^#^: eukaryotic sequences, ^*^: bacteria that have been shown to produce sterols.

In agreement with a previous study, the myxobacterium *C. coralloides* produces significant amounts of squalene but no sterol-like molecules despite having a copy of both squalene epoxidase (SE), required for the conversion of squalene to oxidosqualene prior to cyclization, and Osc in its genome (Table 4 and Figure 6; Bode et al., 2003). To determine if the *C. coralloides* SE and Osc proteins were missing any necessary functional residues, we constructed an alignment of a subset of the bacterial SE and Osc homologs with four eukaryotic SE and Osc sequences (Figure 7; Fischer and Pearson, 2007). Both of these alignments indicate that key functional amino acid positions in the *C. coralloides* SE and Osc proteins are conserved and so the proteins are likely to be functional (Ruckenstuhl et al., 2005; Abe et al., 2007; Fischer and Pearson, 2007). It is also possible that the lack of sterol production may be due a lack of expression under the specific laboratory growth conditions we tested. Current studies are focused on growing *C. coralloides* under various conditions to induce sterol synthesis as well expressing the *C. coralloides* SE and Osc homologs in a heterologous system to verify that these proteins are functional.

### Sterol production in the methanotrophs

**Figure 8:**
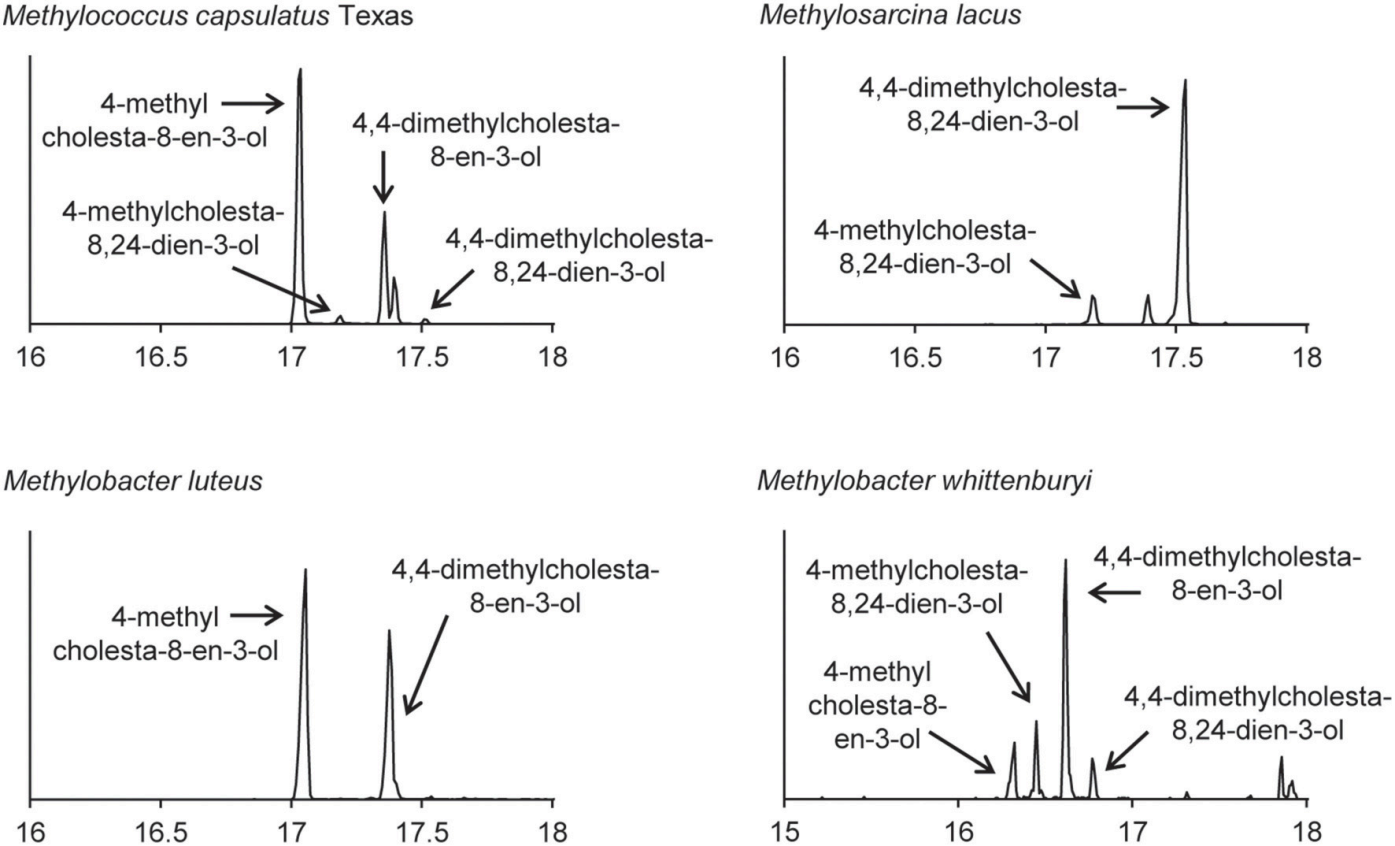
**Sterols production in the aerobic methanotrophs**. Extracted ion chromatograms (m/z 69, 440, 442, 454, 456, 468, and 498) of total lipid extract (TLE) from four aerobic methanotrophs. All TLEs were extracted from liquid cultures and were acetylated prior to running on the GC-MS. Sterol peaks were identified based on their mass spectra as shown in Figure 2.

The lipid profiles of the four Methylococcales species tested were similar to what was previously observed in *M. capsulatus* Bath (Volkman, 2005), with some exceptions (Figure 8). *M. lacus* did not saturate the sterol side chain at C-24 as would be predicted because it lacks a homolog of the C-24(28) sterol reductase (ERG4 in yeast or DHCR24 in humans; Table 4). *M. luteus*, on the other hand, only produced sterols that were saturated at the C-24 position (Figure 8). Interestingly, while all of the Methylococcales tested produced sterols that were partially demethylated at the C-4 position, none had homologs of any of the eukaryotic C-4 demethylase genes (Table 4). These methanotrophs also had sterols in which the unsaturation generated during C-14 demethylation was subsequently removed even though they lack a homolog of the C-14 reductase (ERG24; Table 4). This is in contrast to the previously tested *M. alcaliphilum* (Banta et al., 2015) which does have a homolog of the C-14 reductase (ERG24) indicating that there may be more than one mechanism for this reaction within the Methylococcales.

### Sterol production in other bacterial species

**Figure 9:**
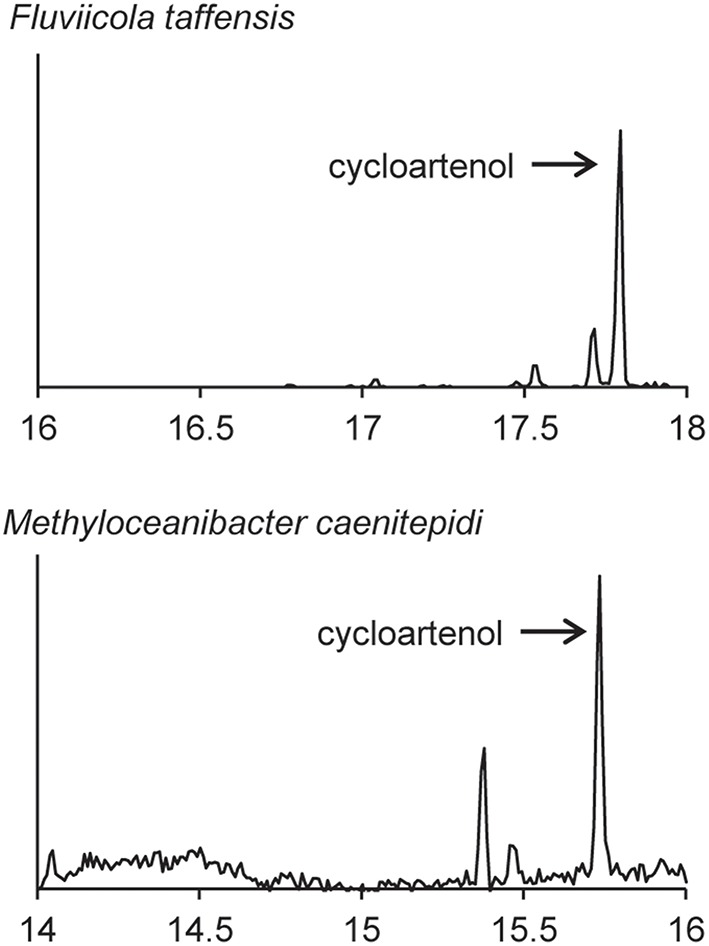
**Sterols production in one Bacteriodetes and one α-Proteobacterium**. Extracted ion chromatograms (m/z 69, 440, 442, 454, 456, 468, and 498) of total lipid extract (TLE) from the Bacteriodetes strains *F. taffensis* and the α-Proteobacterium *M. caenitepidi*. All TLEs were extracted from liquid cultures. The *F. taffensis* TLE was acetylated prior to running on the GC-MS. *M. caenitepidi* TLEs were trimethylsilylated prior to running on the GC-MS. Sterol peaks were identified based on their mass spectra as shown in Figure 2.

We also observed production of cycloartenol in one Bacteriodetes species, *F. taffensis*, and one α-Protebacterium, *M. caenitepidi* (Figure 9 and Table 3). Neither of these strains had homologs of sterol biosynthesis genes downstream of *osc* in their genomes and this was in agreement with our observations of only cycloartenol production (Table 4).

### Cycloartenol vs. lanosterol synthesis is likely correlated with a single residue

The production of cycloartenol by some strains in our survey and lanosterol by others prompted us to investigate if specific residues were indicative of whether a cyclase was a lanosterol or CAS. Site-directed mutagenesis studies have previously identified three amino acids changes that seem to control the product profile of Osc (Meyer et al., 2000, 2002; Lodeiro et al., 2004). Specifically, the amino acid residues T381/C, Q449/V453 (numbering based on human Osc) were indicative of a LAS while Y381/H449/I453 suggested a CAS (Summons et al., 2006). Comparative genomics of three bacterial cyclases with eukaryotic cyclases revealed that only one of these residues was conserved and suggested that a valine (V) or isoleucine (I) at residue 453 suggested lanosterol or cycloartenol production, respectively (Summons et al., 2006). Our lipid analyses and alignments (Figure 7) verify that the bacterial Osc in the organisms we tested completely correlated with the observation that a V453 was indicative of lanosterol production while I453 signified cycloartenol production.

## Discussion

Sterol biosynthesis is primarily viewed as a eukaryotic feature that is rarely observed in the bacterial domain. Here, we coupled bioinformatics with lipid analyses to show that sterol production occurs in diverse bacteria and that this pathway may exist in yet to be discovered bacterial species. Our phylogenetic analysis of one of the key proteins involved in sterol biosynthesis, the Osc, demonstrates that the evolutionary history of this pathway in the bacterial domain is complex. Previous phylogenetic studies have outlined two potential ancestries of sterol synthesis in bacteria—an ancient lineage of LAS and a potentially plant-derived lineage of CAS (Pearson et al., 2003; Desmond and Gribaldo, 2009; Frickey and Kannenberg, 2009). However, these previous phylogenetic analyses were limited as only three or four bacterial Osc sequences were available at the time these studies were undertaken. Our phylogenetic reconstruction with a larger data set demonstrates that Osc homologs fall into two groups—one composed only of bacterial LAS (Group1) and a second composed of both eukaryotic and bacterial Osc sequences (Group 2). The topology of Group 1 does agree with the previous assessment that these sequences may represent a primitive lineage of LAS suggesting that these bacteria possess a more ancestral sterol biosynthetic pathway (Pearson et al., 2003; Desmond and Gribaldo, 2009; Frickey and Kannenberg, 2009; Villanueva et al., 2014). However, the branching of Group 2 sequences brings into question the hypothesis that the second lineage originated through a transfer event from a plant-derived CAS. Several of the bacterial sequences in Group 2 are not CAS and none of them cluster within the Archaeplastida (plant) cyclases. Rather, these bacterial cyclases seem to be forming clades that are distinct from the eukaryotic sequences. One group of bacterial CAS and LAS sequences, however, does branch within the eukaryotic sequences supporting a potential acquisition via horizontal gene transfer from an ancient eukaryote. This distribution of bacterial Osc homologs suggests a complicated ancestry that involves multiple factors including horizontal gene transfer, gene loss, and gene gain. Identification of more bacterial and eukaryotic Osc sequences as well as more rigorous phylogenetic analyses are needed to better interpret the evolutionary history of sterol biosynthesis in both the bacterial and eukaryotic domains.

While our phylogenetic analyses suggest a complex evolutionary history of sterol biosynthesis in bacteria, our lipid analyses demonstrate less modification of sterols in bacteria compared to what is usually observed in eukaryotes. Many of the bacteria we tested produced lanosterol or cycloartenol as the end product. Production of these basic sterols only requires two biosynthetic steps of the canonical eukaryotic sterol pathway—the epoxidation of squalene to oxidosqualene and the subsequent cyclization of oxidosqualene to lanosterol or cycloartenol (Desmond and Gribaldo, 2009). The myxobacteria and methanotrophs, however, did make certain modifications such as C-4 and C-14 demethylations and isomerization of double bonds in the main ring structure. Interestingly, not all proteins required to make those modification in eukaryotes were found in the genomes of these bacteria. In particular, the removal of the C-4 methyl groups requires the activity of three eukaryotic proteins, a C-4 methyl oxidase (ERG25), a C-4 decarboxylase (ERG26) and a C-3 ketoreducatse (ERG27; Bard et al., 1996; Gachotte et al., 1998, 1999). These three proteins were first identified in yeast and homologs have been identified in most sterol producing eukaryotic genomes, with the exception of plants which seem to be missing an ERG27 homolog (Desmond and Gribaldo, 2009). In the myxobacteria, we observed that two of the organisms that removed the C-4 methyl groups had homologs of ERG25 and ERG26 but not ERG27 and a third organism only had a homolog of ERG25. Desmond and Gribaldo attempted to identify potential ERG27 homologs in the genome of *P. pacifica* through comparative genomics (Desmond and Gribaldo, 2009). One potential gene candidate was identified (*P. pacifica* locus tag: Ga0067453_11974) and the myxobacteria we tested do have a homolog of this protein in their genomes. However, further studies are needed to determine if this protein is necessary for C-4 demethylation in the myxobacteria.

It is also possible that downstream sterol modifications in bacteria occur via distinct biochemical pathways than what is observed in eukaryotes. This is a particularly compelling in the aerobic methanotrophs. In these organisms, one methyl group is removed at the C-4 position but we could not identify homologs of the eukaryotic C-4 demethylase genes (ERG25, ERG26, or ERG27). In addition, we observed saturation of the C-14 double bond in the sterols of all methanotrophs tested but did not identify a C-14 reductase (ERG24) in their genomes. The discrepancies in the sterols produced by methanotrophs and the proteins identified in their genomes points to the possibility that novel sterol biosynthesis proteins may exist in bacteria. Identification and characterization of these bacterial sterol proteins could reveal unique biochemical and regulatory mechanisms. In addition, a full understanding of the proteins involved in bacterial sterol production will allow for studies to discern what functional role these lipids play in the bacterial cell and would provide significant insight into the evolution of this ancient biosynthetic pathway.

The lack of significant sterol modifications in bacteria is also noteworthy from a biomarker standpoint. The majority of sterane signatures in the rock record are those that are demethylated at the C-4 positon and/or alkylated at the C-24 positon on the side chain (Summons et al., 2006; Peters et al., 2007b; Love et al., 2009). Our lipid analysis revealed that only some of the myxobacteria fully demethylated at the C-4 position and none of the bacteria surveyed methylated at the C-24 position. While our metagenomic analysis demonstrates that the potential for sterol production exist in yet undiscovered bacteria, we currently do not know the taxonomy of those bacteria nor do we know the chemical structure of the sterols that these bacteria may be producing. Thus, with the knowledge we have at this point, our analyses are supportive of the current interpretation that certain sterane structures in the rock record are indicative of particular eukaryotes. Further, sterane biomarkers are also utilized as proxies for the occurrence of oxygen in ancient environments (Summons et al., 2006; Peters et al., 2007b). In this study, none of the bacteria that were identified to have an Osc homolog in their genome are anaerobes providing strong evidence that sterol synthesis is an aerobic biosynthetic pathway in bacteria like it is in eukaryotes. It is possible that sterols and an alternate sterol synthesis pathway may be discovered in an anaerobic bacterium—as was the case with hopanoids (Hartner et al., 2005; Blumenberg et al., 2006). But again, with the current data available to us, the use of sterane signatures in the rock record as indicators of an oxic environment (at least locally) remains robust.

Finally, this work demonstrates the utility of combining bioinformatics with lipid analyses to obtain a broader picture of not just sterol synthesis in bacteria but potentially other geologically relevant lipids. The increasing amount of genome and metagenome sequence data available along with advancements in culturing and developing genetic systems in non-traditional microbes provides an excellent opportunity for exploring many aspects of biomarker lipids in microbes—their biosynthesis, their function and their evolutionary history. Ultimately, a full understanding of microbial biomarker lipids can provide valuable information to bolster current biosignature interpretations or perhaps allow for more nuanced interpretations of lipid biosignatures in both modern ecosystems and ancient sedimentary rocks.

## Author contributions

JW acquired and analyzed data and was involved in editing the manuscript. XY acquired data and was involved in editing the manuscript. PW designed the study, acquired and analyzed data, and wrote the manuscript.

## Funding

This work was supported by grants from the National Science Foundation (EAR-1418831 and EAR-1451767) to PW.

### Conflict of interest statement

The authors declare that the research was conducted in the absence of any commercial or financial relationships that could be construed as a potential conflict of interest.
